# Professional identity and its associated psychosocial factors among physicians from standardized residency training programs in China: a national cross-sectional study

**DOI:** 10.3389/fmed.2024.1413126

**Published:** 2024-08-29

**Authors:** Zixuan Zeng, Zhanghong Lu, Xiaoping Zeng, Yong Gan, Jiahui Jiang, You Chen, Lei Huang

**Affiliations:** ^1^Department of Psychiatry, Tongji Hospital, School of Medicine, Tongji University, Shanghai, China; ^2^Teaching Office, Renmin Hospital of Wuhan University, Wuhan, China; ^3^School of Medicine, Tongji University, Shanghai, China; ^4^Department of Social Medicine and Health Management, School of Public Health, Tongji Medical College, Huazhong University of Science and Technology, Wuhan, China; ^5^Shanghai Yangpu District Mental Health Center, Shanghai, China; ^6^Department of Medical Education, Tongji Hospital, School of Medicine, Tongji University, Shanghai, China

**Keywords:** China, influential factors, physicians, professional identity, standardized residency training program

## Abstract

**Introduction:**

Shortage and high turnover intention rate of physicians are concerning problems in China. Professional identity has been shown as an influential factor for physicians’ turnover intention. Enhancing physicians’ professional identity in their early phase of career, standardized residency training program (SRTP), may help reduce the turnover rate. This study aimed to investigate the current status of professional identity and explore its associated psychosocial factors among Chinese SRTP trainees, hoping to provide evidence in strengthening the available medical human resources in China.

**Methods:**

The final sample was comprised of 2,267 Chinese SRTP trainees in this cross-sectional survey conducted from 9 March to 20 March in 2023. Descriptive statistics were calculated. Bivariate analyses and hierarchical multiple linear regression were used to analyze potential associated factors of Chinese SRTP trainees’ professional identity.

**Results:**

The average score of respondents’ professional identity was 47.68 (standard deviation, SD = 8.61). Results from hierarchical multiple linear regression analysis showed that being married (*β* = 0.066, *p* < 0.01), having work experience before SRTP (*β* = 0.036, *p* < 0.05), being satisfied with annual income (*β* = 0.062, *p* < 0.01), psychological distress (*β* = −0.144, *p* < 0.001), depersonalization (*β* = −0.053, *p* < 0.05), emotional exhaustion (*β* = −0.380, *p* < 0.001) and resilience (*β* = 0.169, *p* < 0.001) were associated with professional identity (*F* = 114.301, *p* < 0.001). All associated factors can explain 41.1% of the variance in professional identity, and individual psychological variables make up a substantial portion (28.6%) of this influence.

**Discussion:**

Individual psychological variables are strongly associated with professional identity. Helping SRTP trainees reduce psychological distress, alleviate burnout and enhance resilience may be effective ways to promote the formation of their professional identity.

## Introduction

The shortage of physicians has become a pressing challenge on a global scale (1, 2). Escalated healthcare demands, pronounced attrition rates among physicians, and a diminished influx of graduates entering the medical profession have intensified this predicament worldwide (3, 4). In China, there were only 3.04 physicians per 1,000 population in 2022, relatively low compared to many developed countries (5). Physician turnover intention rates range from 10 to 55% (6, 7). More strikingly, almost half (48%) physicians have reported turnover intention since standardized residency training program (SRTP) (8). The cost of physician turnover can be disruptive. It not only exacerbates the scarcity of healthcare personnel, but also impacts the advancement of the medical profession. Research has demonstrated that professional identity is one of the vital influential factors on physicians’ turnover intention, and a stronger professional identity corresponds to a lower inclination to turnover (9, 10).

Based on psychological or cognitive identity theories, identities are considered as cognitive mental representations that reflect an external reality (11). From this standpoint, individuals define who they are (which social group they belong to) through processes of identification, internalisation and comparison within and across social categories (12). These representations are invisible but their content can be discovered by experimental design, structured interviews or questionnaires (13). For physicians, professional identity refers to the self-perception, sense of belonging and identification with the medical profession, which lead them to think, act and feel like a physician (14). Physicians with a strong professional identity tend to have a sense of pride, purpose and dedication to their medical profession, leading to more engagement, satisfaction and fulfillment in their work. Studies have shown that higher levels of professional identity exerts a positive influence on various aspects of personal and occupational development, including the pursuit of advanced medical abilities, stronger sense of responsibilities, discovery of meaning in work, better mental health status, more job satisfaction and less turnover intention (15–17).

Psychological or cognitive identity theories posit that professional identity develops over time and can be influenced by social interactions, though it remains relatively stable (12). The standardized residency training program (SRTP) is a stage when trainees transition form learners into independent physicians (18, 19). Since the sense of professional identity formed in the early clinical practice has a profound influence on their future career path, SRTP is the key phase to improve their professional identity (20, 21). By reinforcing professional identity, it is beneficial for SRTP trainees to form a congruent professional image with their medical vocation. Meanwhile, SRTP trainees are motivated to achieve comprehensive development in professional competence, ethical standards and patient care, thereby preparing them for future independent clinical practice. In order to improve physicians’ professional identity early in this phase, it is essential to investigate and explore the current status and relevant factors of professional identity among SRTP trainees.

Literature has shown that physicians’ professional identity can be affected by demographics and psychosocial factors (22, 23). Physicians with different races, religions, ethnicities, educational backgrounds and socioeconomic statuses reported different levels of professional identity (24, 25). Work-related and individual psychological variables are two significant psychosocial aspects that can influence professional identity (26, 27). Work situations, such as doctor-patient relationships, work experience, work intensity, input and payback, can significantly impact professional identity (28–30). Psychological distress and burnout are prevalent psychological issues among SRTP trainees (31). They have been shown to diminish professional identity and increase the likelihood of turnover (32). Besides, as one of fundamental components of psychological capital, resilience may facilitate their professional identity (33).

To our knowledge, there is a paucity of research investigating the Chinese SRTP trainees’ professional identity nationwide. Despite recognizing the significant impact of psychosocial factors on the formation of professional identity, the specific relationship between them still remains unclear among Chinese SRTP trainees. Therefore, we conducted a cross-sectional survey among this vulnerable population. The aims of this study are: firstly, to provide an overview of the current status of professional identity among Chinese SRTP trainees; secondly, to investigate the relationship between professional identity and psychosocial factors, particularly work-related and individual psychological variables. We anticipate that this knowledge will make a valuable contribution towards enhancing the efficacy of professional identity formation programs and interventions for SRTP trainees. This, in turn, will foster their personal growth, facilitate a smooth transition into the medical profession, and ultimately strengthen the available medical human resources in China.

## Methods

### Study design

We conducted a cross-sectional survey in China mainland from 9 March to 20 March in 2023. Participants in this survey were SRTP trainees in China mainland. The study protocol was approved by the Tongji Hospital of Tongji University Ethics Committee (Registration Number K-W-2023-002).

Convenience sampling was used to collect data. The questionnaire was created at an online survey system.^1^ The link of questionnaire was disseminated to SRTP trainees through managers of SRTP from different provinces and municipalities in China via WeChat group (a widely used social media). After receiving the link, SRTP managers forwarded it to SRTP trainees in their units. All respondents were required to read the participant information sheet and sign an consent form before commencing the questionnaire. In total, 2,364 respondents completed the questionnaire. Those who spent less than 3 min filling the questionnaire were excluded. Finally, 2,267 respondents were enrolled into the analyses, with a validity rate of 95.9%.

Sample size was calculated based on a previous study using the same instrument to measure professional identity of general practitioners (GPs) in China with the average score and standard deviation being 51.23 and 7.06, respectively (34). To set an allowable error of 0.5 and a significance level (*α*) of 0.05, the minimum sample size of 766 was required. The actual sample size was nearly twice more than the required one, thus, it is sufficient for analysis.

### Data collection and quality control

The questionnaire was designed based on literature review and group discussion. The first section collected socio-demographic information. The second section consisted of work-related questions. The third section contained some individual psychological measurement. To improve the quality of questionnaire, a pilot study was conducted by asking 20 SRTP trainees from a particular hospital to complete questionnaires. Based on their feedback, we made adjustments to the questionnaire and implemented a minimum answering time of 3 min as an exclusive criterion. In the process of filling out the questionnaire, all questions were mandatory and participants could terminate at any time. Once all data were collected, two specialized investigators extracted the data from the website database to ensure accuracy.

### Questionnaire measurement

#### Professional identity

The professional identity scale was used to assess SRTP trainees’ professional identity (35). It is derived from the Japanese Nurse’s Career Identity Scale (NCIS), which was developed by researchers at the University of Tokyo and widely used in Japan (36, 37). It consisted of 21 items across seven dimensions: “Sense of Grasp,” “Sense of Consistency,” “Sense of Meaning,” “Self-Efficacy,” “Self-Determination,” “Organizational Influence” and “Patient Influence.” The Cronbach’s *α* for the total scale and each sub-scale ranges from 0.765 to 0.862. In 2010, Zhao et al. translated and validated the scale for measurement of professional identity of nurses in China (38). Subsequently, Wu further simplified and revised the scale to a 13-item version, making it suitable for both Chinese nurses and physicians in 2012 (35). For instance, the item “Nursing work suits me” was adjusted to “Healthcare work suits me.” The Cronbach’s *α* for this revised scale was 0.910. In this study, we utilized the 13-item professional identity scale by Wu, with each item rated on a 5-point Likert scale ranging from 1 (very disagree) to 5 (very agree) (35). The total scores range from 13 to 65 and a higher score indicates a stronger sense of professional identity. For the present sample, the Cronbach’s *α* coefficients and KMO value of this scale were 0.904 and 0.914. Bartlett’s test of sphericity was significant (*χ*^2^ (78) = 15, 568.674, *p* < 0.001). Contents of the scale are presented in Supplementary Table S1.

### Influential factors

#### Demographic characteristics

Demographics included age (continuous variable, years), gender (male or female), marital status (unmarried/divorced or married/cohabitation), region of work (eastern China, central China or western China), highest education level before SRTP (Bachelor’s degree, Master’s degree or Doctoral degree).

#### Work-related variables

Work-related variables included work experience before SRTP (yes or no), years in SRTP (1st, 2nd, or 3rd year), weekly working hours (≥48 h or <48 h), satisfaction with annual income (unsatisfied, neutral, or satisfied) and occupational pressure (low, intermediate or high).

#### Individual psychological variables

Psychological distress was screened by the Patient Health Questionnaire-4 (PHQ-4) which included two subscales: PHQ-2 for depression and GAD-2 for anxiety (39). Each contained two items to assess the frequency (from 0 = not at all to 3 = nearly every day) of depressive or anxious symptoms in the last two weeks. Possible total score of PHQ-4 varied from 0 to 12, with higher scores representing more psychological distress. The Cronbach’s *α* coefficient for PHQ-4 was 0.925.

Burnout was measured using the 2-item version of the Maslach Burnout Inventory (MBI-2), whose items were “I feel burned out from my work” (EE, emotional exhaustion) and “I’ve become more callous toward people since I took this job” (DP, depersonalization). Each item was rated with a 7-point Likert scale ranging from 0 (never) to 6 (every day), with higher scores reflecting greater EE or DP. EE and DP were evaluated separately throughout analyses. West et al. developed this scale and demonstrated its effectiveness in screening the burnout among medical professionals (40).

Resilience was measured by the Connor Davidson Resilience Scale (CD-RISC-2) (41). The CD-RISC-2 included “able to adapt to change” and “tend to bounce back after illness or hardship.” And they were assessed on a 5-point Likert scale ranging from 0 (not true at all) to 4 (true nearly all the time). Total score ranged from 0 to 8, higher suggesting more resilient. In the present study, the Cronbach’s *α* was 0.806.

### Statistical analysis

Descriptive statistics were calculated. Two independent sample *t*-test or one-way ANOVA were used to test the statistical differences in professional identity scores across different subgroups. Pearson’s correlation was used to assess the degree of correlation between age, resilience, psychological distress, burnout (depersonalization and emotion exhaustion) and professional identity scores. Hierarchical multiple linear regression was conducted to examine the associations between selected variables and professional identity. In the first and second blocks, socio-demographics and work-related variables were entered, respectively. In the subsequent three blocks, individual psychological variables including psychological distress, burnout (depersonalization and emotion exhaustion) and resilience were entered in the model subsequently. *P* values less than 0.05 were considered statistically significant. SPSS version 25.0 (IBM Corporation, Armonk, NY, United States) was used for analysis.

## Results

Tables 1, 2 presents the characteristics of respondents. A final sample of 2, 267 respondents were enrolled. 1, 241 (54.74%) were female, and the average age was 26.68 ± 2.97. 23.51, 48.39, and 28.10% worked in western, central and eastern China and the majority (79.44%) were unmarried. More than nine tenths (91.13%) of respondents had a bachelor’s degree, and 52.67% had work experience before SRTP. The proportion of SRTP trainees at the 1st, 2nd and 3rd year were 34.58, 31.36 and 34.06%. Few (10.63%) were satisfied with their annual income. Approximately half (51.35%) worked more than 48 h a week, and over three quarters (75.25%) reported high occupational pressure. The average score for respondents’ professional identity scale was 47.68 (SD = 8.61). The respondents gave a rating score of 4.41 ± 3.34, 2.42 ± 1.95, 2.68 ± 1.96, and 4.72 ± 1.61 for psychological distress, depersonalization, emotion exhaustion and resilience, respectively.

**Table 1:** Descriptive statistics for categorical variables and associations with professional identity.

Variables		*n* (%)	Mean ± SD	*t*/*F*	*p* value
Gender	Female	1,241 (54.74)	47.6 ± 8.0	−0.246	0.806
	Male	1,026 (45.26)	47.7 ± 9.3		
Marital Status	Unmarried/divorced	1801 (79.44)	47.0 ± 8.6	−7.662	<0.001
	Married/cohabitation	466 (20.56)	50.3 ± 8.2		
Region of Work	Eastern China	637 (28.10)	47.5 ± 8.7	5.684	0.003
	Central China	1,097 (48.39)	47.2 ± 8.8		
	Western China	533 (23.51)	48.8 ± 8.1		
Education Level	Bachelor degree	2066 (91.13)	47.6 ± 8.6	1.188	0.305
	Master degree	161 (7.10)	48.6 ± 8.4		
	Doctoral degree	40 (1.77)	48.6 ± 8.4		
Work experience before SRTP	No	1,073 (47.33)	46.8 ± 8.5	−4.389	<0.001
	Yes	1,194 (52.67)	48.4 ± 8.7		
Year in SRTP	1st year	784 (34.58)	47.7 ± 8.5	0.311	0.732
	2nd year	711 (31.36)	47.5 ± 9.1		
	3rd year	772 (34.06)	47.8 ± 8.3		
Satisfaction with annual income	Unsatisfied	1,267 (55.89)	45.6 ± 9.1	112.233	<0.001
	Neutral	759 (33.48)	49.5 ± 7.0		
	Satisfied	241 (10.63)	53.0 ± 7.0		
Weekly working hours	<48 h	1,103 (48.65)	48.3 ± 8.0	3.333	0.001
	≥48 h	1,164 (51.35)	47.1 ± 9.1		
Occupational pressure	Low	41 (1.81)	48.2 ± 8.0	18.811	<0.001
	Intermediate	520 (22.94)	49.7 ± 6.9		
	High	1706 (75.25)	47.1 ± 9.0		

SD, standard deviations; SRTP, standardized residency training program.

**Table 2:** Correlation coefficients of continuous variables and professional identity.

Variables	1	2	3	4	5	6
1. Professional identity	–					
2. Age	0.166^***^	–				
3. Psychological distress	−0.449^***^	0.017^***^	–			
4. Depersonalization	−0.494^***^	−0.134^***^	0.479^***^	–		
5. Emotion Exhaustion	−0.585^***^	−0.118^***^	0.545^***^	0.770^***^	–	
6. Resilience	0.426^***^	0.079^***^	−0.458^***^	−0.370^***^	−0.417^***^	–
Mean	47.68	26.68	4.41	2.42	2.68	4.72
SD	8.61	2.97	3.34	1.95	1.96	1.61

SD, standard deviations; ^***^means *p* < 0.001.

The differences in mean scores of professional identity among subgroups are presented in Table 1. There were significant differences in SRTP trainees’ professional identity across marital status, region of work, work experience before SRTP, satisfaction with annual income, weekly working hours and occupational pressure. Specifically, Those who were married/cohabitation (*t* = −7.662, *p* < 0.001), worked in western China (*F* = 5.684, *p* = 0.003), had work experience before SRTP (*t* = −4.389, *p* < 0.001), were more satisfied with annual income (*F* = 112.233, *p* < 0.001), worked less than 48 h per week (*t* = 3.333, *p* = 0.001) and had more occupational pressure (*F* = 18.811, *p* < 0.001) reported a stronger sense of professional identity. However, no significant differences were detected in professional identity of SRTP trainees by gender, education and year in SRTP (all *Ps* >0.05). In Table 2, professional identity was significantly associated with age, psychological distress, depersonalization, emotion exhaustion and resilience (*r* = 0.166, −0.449, −0.494, −0.585 and 0.426, all *P*s < 0.001).

Table 3 demonstrates the results of the hierarchical multiple linear regression analysis. Marital status, work experience before SRTP, satisfaction with annual income, occupational pressure, psychological distress, depersonalization, emotion exhaustion and resilience were significantly associated with professional identity in the final model with an adjusted *R*^2^ value of 0.411. Specifically, being married (*β* = 0.066, *p* < 0.01), having work experience before SRTP (*β* = 0.036, *p* < 0.05), being satisfied with annual income (*β* = 0.062, *p* < 0.01), and higher level of resilience (*β* = 0.169, *p* < 0.001) were associated with a stronger sense of professional identity. Conversely, higher level of psychological distress (*β* = −0.144, *p* < 0.001), depersonalization (*β* = −0.053, *p* < 0.05) and emotion exhaustion (*β* = −0.380, *p* < 0.001) were associated with a weaker sense of professional identity (*F* = 114.301, *p* < 0.001). Notably, compared with intermediate occupational pressure, low occupational pressure was associated with lower scores of professional identity (*β* = −0.031, *p* < 0.05) and high occupational pressure was associated with higher scores (*β* = 0.047, *p* < 0.05). There was no significant multicollinearity among the variables within the model, as all of the variance inflation factors (VIF) were less than 3. Socio-demographics, work-related variables and individual psychological variables explained 3.5, 9.0, and 28.6% of the variance in professional identity, respectively. Among individual psychological variables, psychological distress, burnout and resilience explained 13.7, 12.9 and 2.0% of the variance in professional identity, respectively.

**Table 3:** Hierarchical multiple linear regression of professional identity.

Variables	Model 1	Model 2	Model 3	Model 4	Model 5
Socio-demographics					
Age (per year)	0.117^***^	0.116^***^	0.097^***^	0.038	0.037
Gender (Female)					
Male	0.002	0.021	0.018	0.027	0.016
Marital status (Unmarried/divorced)					
Married/cohabitation	0.087^**^	0.081^**^	0.093^***^	0.075^***^	0.066^**^
Region of work (Eastern China)					
Central China	−0.014	0.001	0.006	−0.023	−0.018
Western China	0.060*	0.064**	0.042	−0.001	0.006
Education Level (Bachelor degree)					
Master degree	0.004	0.003	0.006	0.004	−0.004
Doctoral degree	−0.007	−0.011	−0.002	0.006	0.005
Work-related variables					
Work experience before SRT (No)					
Yes		0.024	0.037	0.033	0.036^*^
Year in SRTP (1st year)					
2nd year		−0.030	0.000	0.018	0.011
3rd year		−0.046	−0.006	0.035	0.028
Satisfaction with annual income (Unsatisfied)					
Neutral		0.200^***^	0.107^***^	0.039^*^	0.029
Satisfied		0.244^***^	0.162^***^	0.082^***^	0.062^**^
Weekly working hours (<48 h)					
≥48 h		−0.018	−0.002	0.023	0.020
Occupational pressure (Intermediate)					
Low		−0.034	−0.032	−0.026	−0.031^*^
High		−0.070^**^	0.010	0.038^*^	0.047^*^
Individual psychological variables					
Psychological distress			−0.404^***^	−0.195^***^	−0.144^***^
Burnout					
Depersonalization				−0.065^*^	−0.053^*^
Emotion Exhaustion				−0.405^***^	−0.380^***^
Resilience					0.169^***^
Unadjusted *R*^2^	0.038	0.131	0.267	0.396	0.416
Adjusted *R*^2^	0.035	0.125	0.262	0.391	0.411
*F*	12.641	22.536	51.354	81.810	84.347

^*^ means *p* < 0.05; ^**^ means *p* < 0.01; ^***^ means *p* < 0.001.

## Discussion

In this study, the average mean score of professional identity among Chinese SRTP trainees was 47.68 (SD = 8.61). When comparing our results to previous studies conducted on Chinese general practitioners (34), public hospital healthcare workers (35), and blood transfusion physicians (42) using the same measurement, we observe a relatively lower mean score of professional identity among SRTP trainees. Possible explanations for the disparities could be the insufficient experience and role ambiguity. First, SRTP trainees are at the early stages of their medical careers, still navigating the transition from medical school to clinical practice. Lack of experience may impact their confidence in professional roles, as they have less exposure to complex medical scenarios compared to the senior physicians (43). Second, as SRTP involves rotations across various medical settings, trainees may still be in the process of exploring different specialties and determining specific professional goals (44). This lack of role clarity could contribute to a lower level of professional identity (45).

According to the social-cognitive approach by Berzonsky, professional identity is influenced by cognitive processes such as reflection, evaluation and regulation in identity construction, and associated with some social relationships and environments (11). Consistent with this theory, findings are that professional identity is significantly associated with social factors including marriage, work experience, annual income, occupational pressure, and psychological factors including psychological distress, depersonalization, emotional exhaustion and resilience, all of which may affect cognitive process.

Being married, having working experience before SRTP, being satisfied with income were positive associated factors of SRTP trainees’ professional identity. Firstly, marriage may provide them with a sense of stability, emotional support and belonging, which in turn motivates them to work diligently and enhances their professional identity (46). Secondly, trainees with previous working experience were likely to possess a better understanding of their chosen career path, have enhanced professional skills, and thus exhibit a stronger professional identity (43). Thirdly, satisfaction with income was found to have a positive influence on professional identity. But it was noticeable that 55.89% of SRTP trainees in our study were unsatisfied with their annual income, consistent with the findings of our earlier investigation among SRTP trainees in Shanghai (47). In fact, SRTP trainees in China undertook significant workload within the hospital setting, yet they continued to face financial constraints (48). The discrepancy between demanding responsibilities and low income can have negative implications for their professional identity (49). Therefore, supporting trainees with adequate financial security enables them to dedicate themselves fully to their professional roles and pursue their aspirations.

Notably, in the univariable analysis, SRTP trainees with “intermediate occupational pressure” had the highest professional identity score. However, in the multivariable analysis, the score of “intermediate occupational pressure” was higher than that of “low occupational pressure,” lower than that of “high occupational pressure.” These results both indicated that moderate work stress might benefit the formation and development of professional identity. A plausible explanation could be that resident physicians who set higher self-expectations in their work and exhibit greater work engagement may have higher stress level, which will meantime promote professional identity formation (34). But the inconsistency between univariable and multivariable analyses may be due to interaction effects between influencing factors. Future research is needed to delve into the interactions between occupational pressure, individual psychological factors and professional identity. Besides, since we assessed occupational pressure using subjective self-assessment through a three-category stress rating rather than utilizing validated and standardized scales specifically designed to measure stress, this finding should be interpreted with caution.

Psychological distress was significantly negatively associated with professional identity among Chinese SRTP trainees, in accordance with the findings reported by Adisaputri et al. (50). A reason for this might be that SRTP trainees with more psychological distress may harbor more negative self-evaluations such as feeling inadequate or incompetent. These negative self-perceptions can hinder the development and maintenance of their professional identity (51). Another possible interpretation is that higher levels of psychological distress often coincide with reduced work enthusiasm and engagement (52). SRTP trainees with depression and anxiety may struggle to sustain their passion for work and become disengaged from their professional roles, ultimately resulting in a weakened sense of professional identity (51). Furthermore, compromised mental health among SRTP trainees can lead to adverse medical outcomes, such as medical errors or lower-quality care (53). As professional identity is closely intertwined with the delivery of high-quality care and feelings of fulfillment in their role as caregivers, psychological distress could exert a detrimental impact on professional identity (54). In light of this, psychological support for trainees can be enhanced by offering peer support programs that foster a sense of belonging and one-on-one counseling sessions that provide personalized assistance (55).

As crucial components of the burnout syndrome, higher levels of emotional exhaustion or depersonalization was significantly associated with lower levels of professional identity among SRTP trainees. This has been evidenced among Portugal healthcare workers (56), Chinese emergency physicians (57) and primary health workers (58). Several plausible explanations can account for these results. Firstly, emotional exhaustion induces fatigue and diminish emotional engagement, making it more challenging for trainees to experience fulfillment and satisfaction in their professional roles, which are crucial components of professional identity (59). Moreover, trainees with elevated emotional exhaustion may have less empathy and compassion towards patients and become disillusioned with their profession (60). This may prompt them to question their career choices and contemplate alternative paths, potentially leading to a weakened sense of professional identity and even increasing the likelihood of turnover (8). When depersonalization occurs, where SRTP trainees display cynicism or treat patients as objects rather than individuals with unique needs, it creates a barrier to effective communication and trust between doctor and patient. Given that professional identity is closely linked to the ability to establish meaningful connections with patients and deliver patient-centered care, depersonalization serves as an impediment to develop professional identity (61). Balint Group, developed by Michael and Enid Balint in the 1950s, is a highly recommended activity to be held regularly to prevent burnout, process identity information and deal with identity conflicts (62, 63). It provides resident physicians with a safe, confidential and non-judgmental space to fully share their thoughts, confusions and emotions about a particular clinical dilemma, encouraging individuals to explore their professional beliefs, values, and goals, which may not be highlighted in daily clinical practices and regular training curricula (64).

Resilience was found to be a positive association of professional identity, concurring with the former results (65). Resilience can enable physicians to adapt, cope and bounce back from challenges, setbacks, and stressors (66). SRTP trainees may generally encounter various stressors such as limited clinical skills, financial constraints, demanding academic workload, family responsibilities, and fierce job competition (48). Resilience thus helps them to overcome these challenges that lead to identity conflicts. For SRTP educators, they can integrate narrative medicine (NM) into the SRTP medical education. NM is a scientifically validated, effective educational intervention that has been shown to increase physicians’ resilience, empathy, affinity, professionalism (67, 68). It can facilitate professional identity by guiding SRTP trainees in self-exploration, fostering critical thinking, promoting reflective learning and increasing understanding of patients (69). However, it is rarely applied among Chinese SRTP trainees.

To our knowledge, this study represents one of the earliest investigations into the status of professional identity and its associated factors among Chinese SRTP trainees. Additionally, the study offers insights into the psychosocial factors that may impact the professional identity of SRTP trainees, thereby providing evidence to support the implementation of tailored interventions for cultivating their professional identity. However, this study has several limitations. Firstly, being a cross-sectional study, it is limited in establishing causal relationships between variables. Secondly, the online data collection through self-filled questionnaires may introduce response biases, as participants who were more enthusiastic and optimistic about their work might be more likely to complete the questionnaire. Thirdly, validated and standardized scales of occupational pressure should be used to explore its relationship with professional identity and the potential interactions with other psychological factors. Future research should consider implementing a longitudinal design to examine the dynamic nature of professional identity over time and explore the temporal relationship between psychosocial factors and professional identity among SRTP trainees.

## Conclusion

This study indicates that individual psychological factors, namely psychological distress, burnout and resilience may have a substantial influence on professional identity, explaining 28.9% of the variance. Specifically, higher levels of psychological distress and burnout are associated with weaker senses of professional identity. Instead, more resilience may promote professional identity. It is imperative for managers and educators to develop tailored interventions and measures to help SRTP trainees reduce psychological distress, alleviate burnout and enhance resilience, which will help promote professional identity and thereby reduce the turnover rates and strengthen the available medical human resources in China.

## Data Availability

The raw data supporting the conclusions of this article will be made available by the authors, without undue reservation.

## Footnotes

^1^ https://www.wjx.cn
