# Supplementary material for: Professional identity and its associated psychosocial factors among physicians from standardized residency training programs in China: a national cross-sectional study
Source: Front Med (Lausanne). 2024 Aug 29;11:1413126. doi: 10.3389/fmed.2024.1413126 (PMC11390412; doi:10.3389/fmed.2024.1413126)
Supplement: Supplementary file 1 [file Table_1.DOCX]

**Table 1** Items of professional identity scale

| Items |
| --- |
| 1. When referring to my profession, I usually say “we” rather than “they”. |
| 1. I consider my success as the success of healthcare workers. |
| 1. I care very much about other people’s views on my career. |
| 1. When others praise my profession, it feels like a personal compliment to me. |
| 1. If there are some criticisms on my career from the media, I will feel ashamed and embarrassed. |
| 1. My job is important. / I believe in the importance of my job. |
| 1. I am confident in my ability on work. |
| 1. The work I do affects the condition of my patients. |
| 1. My job is meaningful. |
| 1. I have the necessary qualifications and skills for my job. |
| 1. I understand the responsibilities and requirements of the work. |
| 1. Healthcare work suits me. |
| 1. I know my role. |
